# Aspirin loading in coronary artery disease patients already taking aspirin: A systematic review

**DOI:** 10.34172/jcvtr.025.33481

**Published:** 2025-09-28

**Authors:** Hila Asham, Ahmad Separham, Mohammad Javad Kamali, Musab Hama Faraj, Mehdi Maleki, Maryam Mehrpooya, Parvin Sarbakhsh, Taher Entezari-Maleki

**Affiliations:** ^1^Department of Clinical Pharmacy, Faculty of Pharmacy, Tabriz University of Medical Sciences, Tabriz, Iran; ^2^Cardiovascular Research Center, Tabriz University of Medical Sciences, Tabriz, Iran; ^3^Department of Clinical Pharmacy, School of Pharmacy, Hamadan University of Medical Sciences, Hamadan, Iran; ^4^Department of Statistics and Epidemiology, Faculty of Public Health, Tabriz University of Medical Sciences, Tabriz, Iran

**Keywords:** Aspirin, Reloading, Coronary artery diseases, Thromboxane B_2_, Percutaneous coronary intervention

## Abstract

Aspirin is considered a cornerstone medication among patients with established coronary artery disease (CAD). There is a lack of evidence regarding aspirin reloading in CAD patients who are already receiving aspirin therapy. We performed this systematic review to address this gap of knowledge. A systematic review on PubMed, Embase, and the Cochrane Library was conducted from inception until July 15, 2024. Two authors independently performed study selection, data extraction, and risk of bias assessment. Means differences (MD) were used in a meta-analysis of related outcomes from the studies. Our review included four studies enrolling 1187 individuals with CAD and chronic aspirin use before admission. The results of this systematic review found that aspirin reloading is significantly associated with a reduction of thromboxane B_2_ (MD, -17.46; 95% CI, -19.61 to -15.32; *P*<0.00001; I^2^=0%). Additionally, our findings revealed the beneficial effects of aspirin loading on thromboxane B_2_ -related platelet reactivity and myocardial injury indexes. No significant adverse outcomes, such as bleeding and increased mortality, were observed among the study groups. In conclusion, aspirin reloading can improve cardiovascular outcomes with a good safety profile among CAD individuals. However, further randomized clinical trials (RCTs) are still needed to provide robust evidence.

## Introduction

 Aspirin, an irreversible inhibitor of cyclooxygenase-1 (COX-1), is a cornerstone treatment for most patients with established coronary artery disease (CAD) to avoid further cardiovascular events.^1^

 The potential benefits of long-term aspirin therapy as secondary prevention have been previously established by the Antithrombotic Trialists’ Collaboration group.^2^ A meta-analysis of 195 randomized trials of antiplatelet therapy with aspirin, involving more than 135,000 high-risk patients with prior cardiovascular disease (CVD) showed that aspirin significantly reduced the risk of subsequent vascular events, such as nonfatal myocardial infarction (MI), nonfatal stroke, and death.^3^

 Aspiring loading (either in chewable form or intravenously) upon initial presentation of acute coronary syndrome (ACS) symptoms is strongly recommended as class I, according to the latest guidelines of coronary artery revascularization.^4,5^ The similar recommendation is also consistent for individuals with stable ischemic heart disease (SIHD) undergoing elective percutaneous coronary intervention (PCI).^5^

 Although the new 2025 ACS guideline mentioned reloading of aspirin in patients already taking aspirin, there is still a lack of evidence to strongly recommend this issue.^5^ Therefore, we aimed to perform a systematic review of the literature to provide robust evidence regarding aspirin reloading in patients already taking aspirin. To the best of our knowledge, this is the first systematic review of aspirin reloading in this particular gap of evidence.

## Methods

 This study protocol was registered in the International Prospective Register of Systematic Reviews (PROSPERO) with ID: CRD42024571230 and was in accordance with the Preferred Reporting Items for Systematic Reviews and Meta-Analyses (PRISMA 2020 Checklist).^6^ The full checklist is available in Table S1.

###  Data Sources and Search Process

 A systematic search of electronic databases (PubMed, Embase, and the Cochrane Collaboration Central Register of Controlled Trials) was independently performed by two authors (H.A. and M.K.) from the earliest publication date available until July 15, 2024. Additional search was also performed from Google Scholar. The relevant keywords used were: ‘(“Aspirin” OR “ASA” OR “Acetylsalicylic Acid”) AND (“Percutaneous Coronary Intervention” OR” Percutaneous Coronary Revascularization” OR “Myocardial Infarction” OR “Angina” OR “Acute Coronary Syndrome” OR “Coronary Artery Diseases”) AND (“Reload” OR “Loading Dose”)’. The detailed information is reported in Table 1. A rigorous screening process was employed to determine the relevance of the search results.

**Table 1:** The detailed search strategy for investigated databases

**Database**	**Search strategy**		**No. of results**
	**#Search No.**	**Query**	
PubMed	#1	"Aspirin"[Title/Abstract] OR "ASA"[Title/Abstract] OR "Acetylsalicylic Acid"[Title/Abstract]	94155
	#2	"Reload"[Title/Abstract] OR "loading dose"[Title/Abstract]	8232
	#3	"Percutaneous Coronary Intervention"[Title/Abstract] OR" Percutaneous Coronary Revascularization"[Title/Abstract] OR "myocardial infarction" [Title/Abstract]OR "angina"[Title/Abstract] OR "acute coronary syndrome"[Title/Abstract] OR "coronary artery diseases" [Title/Abstract]	294153
	#4	#1 and #2 and #3	346
Cochrane library	#1	Aspirin OR ASA OR "Acetylsalicylic Acid" in Title Abstract Keyword - (Word variations have been searched)	53306
	#2	"reload" OR "loading dose" in Title Abstract Keyword - (Word variations have been searched)	6719
	#3	"Percutaneous Coronary Intervention" OR "Percutaneous Coronary Revascularization" OR "myocardial infarction" OR "angina" OR "acute coronary syndrome" OR "coronary artery diseases" in Title Abstract Keyword - (Word variations have been searched)	65541
	#4	#1 and #2 and #3	621
Embase	#1	Aspirin:ti,ab OR ASA:ti,ab OR 'Acetylsalicylic Acid':ti,ab	8578918
	#2	reload:ti,ab OR 'loading dose':ti,ab	5844
	#3	'Percutaneous Coronary Intervention':ti,ab OR 'Percutaneous Coronary Revascularization':ti,ab OR 'myocardial infarction':ti,ab OR angina:ti,ab OR 'acute coronary syndrome':ti,ab OR 'coronary artery diseases':ti,ab	74886
	#4	#1 and #2 and #3	467

 All the retrieved studies were imported to the EndNote version 21.3 and after removing duplications, both authors reviewed the titles and abstracts of the results to identify relevant literature. Following this, full texts of the retained results were evaluated independently, by pre-determined criteria based on Population, Intervention, Comparison, and Outcome (PICO) framework. The references of retrieved articles were screened for further relevant studies. Any discrepancies were resolved through discussion, with a requirement for full agreement before including a study. If necessary, the corresponding author was contacted to settle any remaining disagreement.

###  Study Selection

 Clinical studies eligible for consideration included those that, enrolled participants with CAD, who were already on maintenance aspirin therapy. Additionally, the studies should compare a reloading dose of aspirin at any dose with no aspirin loading and provide information on primary and secondary cardiovascular outcomes and bleeding. Furthermore, the studies were required to be published in the English language. Exclusion criteria for our literature search include articles that involve animal experiments, case studies, practice guidelines, reviews, book chapters, and editorials.

###  Data Extraction

 The data extraction report includes a comprehensive summary of the study setting, methodology, and results. The report includes the following key information: study setting, first author’s last name, year of publication, baseline participant characteristics (total number of participants, mean age, sex, comorbidities, and other relevant demographic data), inclusion criteria, study intervention and control treatment (dose, route of administration), follow-up duration, and endpoint data (outcome name, time measurement, assumed risk estimates, and significance).

###  Quality Assessment 

 Risk of bias assessment was conducted independently by two authors using the Cochrane Collaboration risk of bias tools of the RoB 2 and the ROBINS-I for randomized clinical trials (RCTs) and observational studies, respectively.^7,8^ Risk of bias plots for the included studies were created using the robvis tool for visualizing risk of bias assessments in systematic reviews. Any disagreements were resolved through discussion with the corresponding author.

###  Data Synthesis

 We performed data analysis using MedCalc statistical software version 19.5.3 and RevMan (Copenhagen, The Nordic Cochrane Centre) version 5.3. The results were adjusted to means and standard deviations (SD) for each study. This meta-analysis estimated the mean difference (MD) as the study outcome with a 95% confidence interval (95% CI) using both random-effects and/or fixed-effects models. In cases where statistically significant heterogeneity was found, we implemented a random-effects model for reporting effect sizes.

 A *P* value of < 0.05 was considered statistically significant in our analysis. The forest plot presents all effect sizes and CIs (95%), Tau-squared (Tau2), Chi-squared (Chi2), degree of freedom (df), and *P* value for more details. Statistical heterogeneity between studies was assessed using I^2^ statistics. I^2^ index suggests that 0% to 25% heterogeneity is considered not important, 25% to 50% is moderate, 50% to 75% is substantial, and 75% to 100% is considerable.^9^

## Results

###  Study Selection 

**Figure 1:**
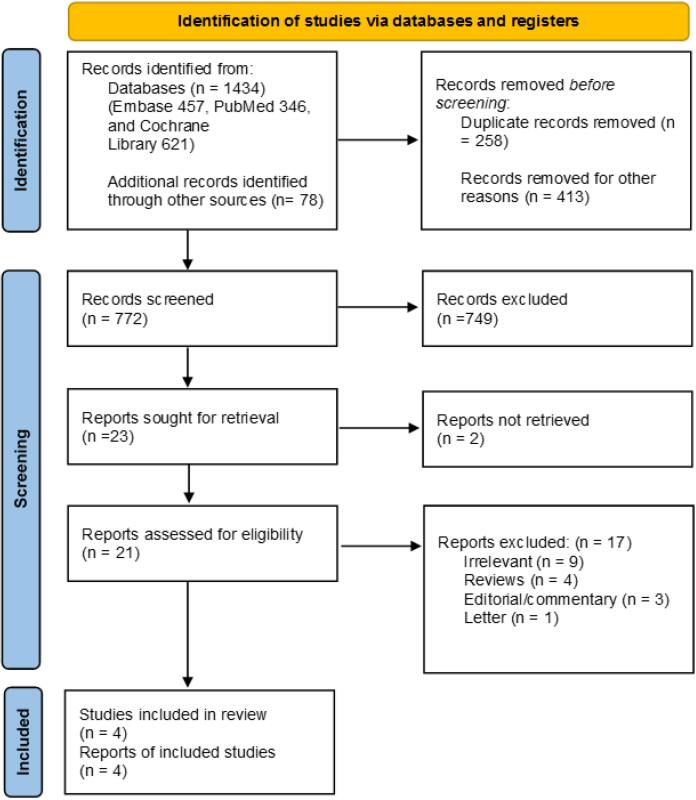
PRISMA 2020 flow diagram of systematic review

 In this systematic review, 1434 studies were obtained through a literature search. Of these, 772 studies were entered after duplication and initial removal. Then, 749 records were excluded based on title and abstract screening, and 23 studies were included. Of 21 retrieval studies, 17 articles were excluded for the following reasons; irrelevant (n = 9), reviews (n = 4), editorial/commentary (n = 3), and letter (n = 1). Four studies (three observational studies, and one RCT) were eventually included in the systematic review.^10-13^ Figure 1 demonstrates the PRISMA flow diagram for the systematic review.

###  Risk of Bias Assessment Results

**Figure 2:**
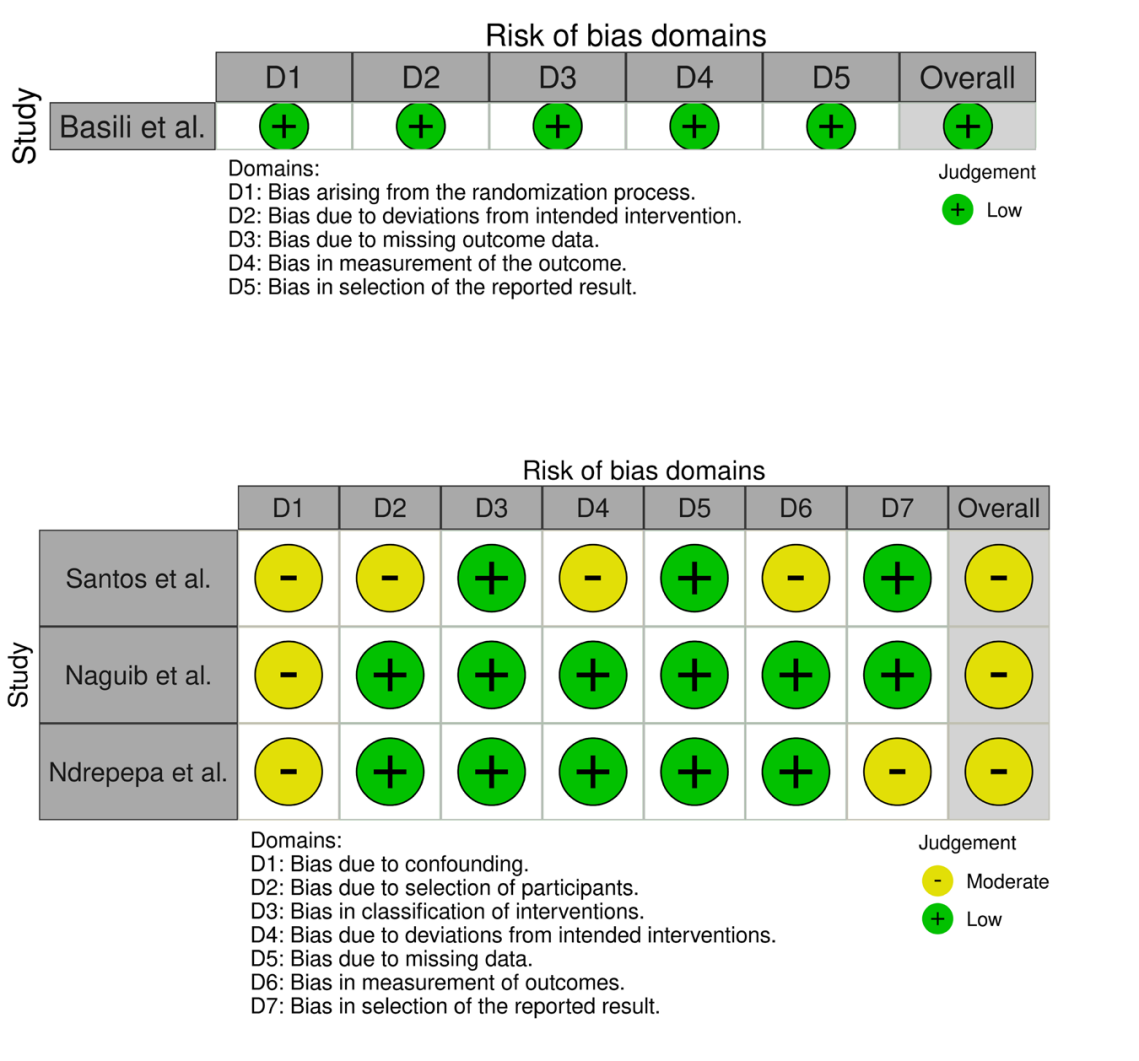
Risk of bias assessment for studies included in this systematic review

 The results of the ROBINS-I tool for observational and RoB-2 for one RCT are shown in Figure 2. Figure 2 also displays the result of the robvis visualizing tool.

###  Study Characteristics

 Overall, 1187 patients across 4 studies were entered.^10-13^ The mean age of participants ranged from 65.8 to 72 years old, with a 75% male population. Different doses of aspirin were used as loading doses in the studies (ranging from 200 to 500 mg). All included patients received dual antiplatelet therapies (DAPT), and no significant difference was observed between the two groups. Two studies (Santos et al^11^ and Basili et al^10^) utilized the same DAPT regimen, including aspirin and clopidogrel. In Ndrepepa’s study,^13^ DAPT therapy included aspirin and ticagrelor or prasugrel. Naguib et al^12^ patients also received DAPT therapy, including aspirin and a P2Y12 inhibitor. The studies were conducted in Germany (n = 2), Spain (n = 1), and one multicenter study in Italy. The characteristics and outcomes of the included studies are presented in Tables 2 and 3, respectively.

**Table 2:** Characteristics of the studies included in the systematic review

**Study, Year**	**Location **	**Setting **	**Patients **	**Age; mean±SD (years)**	**Male (%)**	**Intervention **	**Control **	**Other treatments**
Santos et al 2013^11^	Spain	Observational study	Patients with AMI who were on chronic aspirin therapy	Intervention: 67.14 ± 12 Control: 71.93 ± 11	71	Reloading of aspirin (200–500 mg) (49 patients)	No reloading (15 patients)	ACEi, beta-blockers, statin, CCB, nitrate, aspirin, enoxaparin, fibrinolysis, clopidogrel
Basili et al 2014^10^	Multicenter/ Italy	Double-blind, placebo-controlled RCT	Patients with CAD who were on chronic aspirin therapy undergoing PCI	Intervention: 66 ± 8 Control: 66 ± 12	81.31	Reloading of aspirin (325 mg orally) (46 patients)	No reloading (45 patients)	ACEi / ARB, beta-blockers, CCB, statin, insulin, oral hypoglycemic agents, aspirin, clopidogrel
Naguib et al 2021^12^	Germany	Observational study	Patients with CAD who were on chronic aspirin therapy undergoing PCI	Intervention: 72 ± 10 Control: 72 ± 11	68	Reloading of aspirin (500 mg IV) (50 patients)	No reloading (50 patients)	ACEi /ARB, aldosterone antagonists, beta-blockers, nitrate, statins, diuretics, PPIs, aspirin, phenprocoumon, digitalis, oral antidiabetics, insulin, P2Y12 inhibitors
Ndrepepa et al 2024^13^	Germany	Observational study	Patients with AMI who were on chronic aspirin therapy undergoing PCI	Intervention: 65.8 ± 12.0 Control: 64.1 ± 12.1	76	Reloading of aspirin (150–300 mg oral or chewed or IV) (788 patients)	No reloading (144 patients)	Aspirin, ticagrelor, prasugrel

Abbreviations: ACEi, angiotensin-converting enzyme inhibitors; AMI, acute myocardial infarction; ARB, angiotensin II receptor antagonist; CAD, coronary artery diseases; CCBs, calcium channel blockers; CK, creatine kinase; IV, intravenous; PCI, percutaneous coronary intervention; RCT, randomized clinical trial

**Table 3:** Outcomes of included studies in the systematic review

**Study, Year**	Outcome of interest^*^	**Adverse events**	**Risk of bias **
Santos et al 2013^11^	Reduction in Thromboxane B_2_: significant Reduction C-5HT release: significant Reduction AA-induced platelet aggregation: significant PFA-100 occlusion time assay with CEPI cartridges prolonged: significant ADP: non-significant TRAP-induced aggregation: non-significant Closure time in the PFA-100 assay with CADP cartridges: non-significant	Not reported	Low
Basili et al 2014^10^	Reduction in Thromboxane B_2_: significant Reduction in cTFC end of the PCI: significant Reduction in cTnI end of the PCI: significant Improvement in LVEF level: significant Improvement in myocardial blush grade: significant	Mortality: non-significant Thrombosis-related events: non-significant Bleeding: non-significant	Some concerns
Naguib et al 2021^12^	AA-induced MoA: non-significant Rate of HTPR to aspirin: non-significant	MACCE: non-significant TIMI minimal bleedings: non-significant TIMI major bleedings: non-significant	Some concerns
Ndrepepa et al 2024^13^	Not reported	Higher risk of CNR: significant	Some concerns

Abbreviations: AA, arachidonic acid; ADP, adenosine diphosphate; C-5HT, collagen-induced 5-hydroxytryptamine; CADP, collagen and ADP coated cartridges; CEPI, collagen and epinephrine coated; CNR, coronary no reflow; cTFC, corrected thrombolysis in myocardial infarction frame count; cTnI, cardiac troponin I; HTPR, high on-treatment platelet reactivity; PCI, percutaneous coronary intervention; PFA-100, platelet function analyzer; MACCE, major adverse cerebro and cardiovascular events; MoA, maximum of aggregation; TIMI, thrombolysis in myocardial infarction; TRAP, thrombin receptor agonist peptide device ^*^All outcomes were reported as effects of loading dose of aspirin on intervention group compared to the control group

###  Impact of aspirin reloading on thromboxane B_2_

**Figure 3:**
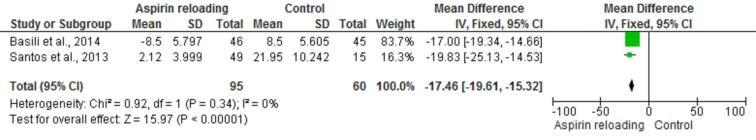
Impact of aspirin reloading on thromboxane B_2_ level

 The effects of aspirin reloading on thromboxane B_2_ among individuals on maintenance aspirin therapy with CAD were evaluated in two studies.^10,11^ The meta-analysis of these studies demonstrated that aspirin reloading could significantly reduce the level of thromboxane B2 from baseline compared to the individuals who only continued a daily dose of aspirin (MD, -17.46; 95% CI, -19.61 to -15.32; P < 0.00001; I^2^ = 0%). The result is shown in Figure 3.

###  Impact of Aspirin Reloading on Platelet Activity

 Aspirin reloading among patients with a maintenance dose of aspirin before admission had some favorable effects on platelet activity other than thromboxane B_2_.^10,13^ Santos et al conducted a non-randomized, observational study to investigate the impact of loading dose of aspirin (200-500 mg) on patients presenting with acute MI while receiving chronic aspirin treatment on platelet reactivity.^11^ The results of this study revealed that administration of a loading dose of aspirin significantly reduced collagen-induced-5HT release, arachidonic acid (AA)-induced aggregation, and prolonged the platelet function analyzer device PFA-100 occlusion time with collagen and epinephrine coated (CEPI) cartridges compared to patients treated with 100 mg/day of aspirin (all *P* < 0.01).^11^ This suggests a marked downregulation of COX1-dependent platelet reactivity in patients who received a loading dose. However, it appears that the loading dose of aspirin provided no significant impact on both adenosine diphosphate (ADP) and thrombin receptor agonist peptide (TRAP)-induced platelet aggregation or the closure time in the PFA-100 assay with collagen and ADP-coated (CADP) cartridges.^11^

 A recent study conducted by Naguib et al aimed to investigate the rate of high on-treatment platelet reactivity (HTPR) to aspirin in patients undergoing elective PCI who received an additional loading dose of aspirin while receiving their daily maintenance aspirin compared to those who did not receive a loading dose.^12^ The study found that the AA-induced maximum of aggregation (MoA) was not significantly different between the intervention and control groups, (Intervention vs. control: 7.0% ± 9.8 vs. 12.9% ± 21.1%; P = 0.24) Furthermore, the rate of HTPR (defined as > 20% MoA) to aspirin did not differ significantly between the two groups (Odds ratio, 0.33; 95% CI, 0.08 to 1.35; *P* = 0.12).^12^

###  Impact of Aspirin Reloading on Cardiac Outcomes

 A multicenter double-blinded RCT evaluated the effects of aspirin loading (325 mg orally) among patients with CAD who were on low-dose chronic aspirin therapy (100 mg daily) undergoing PCI compared to patients who didn’t receive the aspirin loading dose.^10^ Before PCI, the mean corrected thrombolysis in myocardial infarction frame count (cTFC) and cardiac troponin I (cTnI) were similar between the two groups. At the end of the procedure, cTFC and cTnI were significantly lower in the aspirin reload group compared to the control group (P = 0.0023) and (P = 0.046), respectively. The results of this study also demonstrated that 61% of patients allocated to receive aspirin loading therapy were able to reload and achieve normal microcirculatory reperfusion, as measured by myocardial blush grade 3 after the PCI procedure. In contrast, only 32% of patients in the control group reached this level of reperfusion (*P* = 0.0067).^10^

 In addition, the left ventricular ejection fraction (LVEF) values of the control group did not change significantly 72 hours after PCI. However, the aspirin reload group displayed a statistically significant increase in LVEF values, from 50 ± 9% at baseline to 53 ± 7% after 72 hours. Notably, there was a significant difference between the two groups in terms of the change in LVEF values from baseline (-1.95 vs. + 3.15%; *P* < 0.001).^10^

###  Aspirin Reloading Adverse Outcomes 

 Despite the promising effects of aspirin reloading, some adverse events have been reported in included studies. A recent study by Ndrepepa et al revealed that among 932 patients with acute MI undergoing PCI and being chronically treated with a daily dose of aspirin, a single oral or intravenous dose of 150-300 mg aspirin as a loading dose was associated with a higher risk of coronary no-reflow (CNR) compared to patients who only continued their daily aspirin dose. (4.88% vs. 1.05%; OR, 4.85;1.38 to 17.01; *P* = 0.014).^13^

 Considering the risk of bleeding, Naguib et al demonstrated that thrombolysis in myocardial infarction (TIMI) minimal bleeding did not differ among individuals who were treated with aspirin loading and who were not. (odds ratio, 0.15; 95% CI; 0.02 to 1.30; *P* = 0.08). The results of this study did not show a significant difference in major adverse cerebrovascular and cardiovascular events (MACCE) or TIMI major bleeding between study groups.^12^

 The results of Basili et al reported no adverse events during hospitalization in both groups. Additionally, no mortality and thrombosis-related events were observed after a mean follow-up of 12 ± 4 months.^10^ Moreover, no major or minor bleeding occurred during and after the PCI.

## Discussion

 Our current systematic review aimed to provide evidence regarding the potential benefits of aspirin loading among individuals who were already on maintenance daily aspirin use. Notably, there is limited evidence available regarding the effect of aspirin loading in this setting, which suggests a gap in current guidelines. To the best of our knowledge, this is the first systematic review evaluating this important knowledge gap.

###  Impact of Aspirin Reloading on Platelet Reactivity

 Thromboxane B_2_, a stable and inactive metabolite of thromboxane A_2_, has a crucial role in vasoconstriction and platelet aggregation.^14^ A recent study involving 3,044 participants found a significant association between thromboxane B_2_ levels and all-cause and cardiovascular mortality, regardless of aspirin use.^15^ The study’s findings demonstrated that individuals with the upper thromboxane B_2_ quartiles had a 96% increased risk of all-cause mortality and a 141% increased risk of cardiovascular mortality compared to lower quartiles.^15^ Notably, a meta-analysis of two studies (Basili et al and Santos et al)^10,11^ in our literature revealed that aspirin loading is associated with a significant reduction in thromboxane B_2_ levels among individuals with CAD who were on maintenance aspirin prior to admission, compared to the control group. It was worth mentioning that, a previous RCT found that chronic treatment with a 100 mg aspirin/day failed to inhibit the immediate increase in serum thromboxane B_2_ levels following elective PCI.^16^ This highlights the importance of aspirin reloading in this setting. The results from Santos et al observational study also demonstrated significant inhibition of thromboxane B2-dependent platelet reactions, including serotonin release, AA-induced aggregation, and thrombus formation time evaluated by PFA-100.^11^ In contrast, Naguib et al observational pilot study from our systematic review did not show a significant impact or benefit of additional aspirin loading in patients on maintenance aspirin medication undergoing elective PCI in terms of the rate of HTPR to aspirin and AA-induced mechanism of action as pharmacodynamics responses.^12^

###  Impact of Aspirin Reloading on Myocardial Reperfusion

 Loading dose of aspirin before PCI and following ACS received the level I recommendation in ACC/AHA guidelines.^5^ Recently, a retrospective cardiac magnetic resonance imaging (CMR) study, showed that a loading dose of 500 mg oral aspirin prior to primary PCI (PPCI) among 78 patients significantly improved left ventricular (LV) contractility, reduced myocardial damage and minimized early LV adverse remodeling, in which following the loading dose.^17^ However, the effect of aspirin loading among patients who were on chronic aspirin therapy is unclear.

 Basili et al conducted a RCT on the effects of aspirin reloading before elective PCI and showed a higher rate of normal microcirculatory reperfusion among patients who received reloading of aspirin compared to those who did not.^10^ The study also found a notable reduction in cTFC and cTnI levels after PCI, indicating improved myocardial reperfusion and reduced myocardial injury. Furthermore, the findings revealed a significant improvement in LVEF levels 72 hours after PCI for patients who received aspirin reloading, compared to the control group. These findings are in line with previous studies that have shown the beneficial effects of aspirin loading on myocardial injury indexes.^10^

###  Adverse Events of Aspirin Reloading

 Based on the results of a previous study on 12,562 patients with ACS using aspirin chronically at daily doses of 75 to 325mg, a dose-related adverse event of bleeding risk was observed, with higher rates identified in doses exceeding 200mg.^18^ However, our systematic review did not find a significant risk of major or minor bleeding associated with loading doses of aspirin in chronic low-dose aspirin therapy among patients with CAD.^10,12^ This may be due to the fact that a single high-dose loading of aspirin is not inherently associated with a higher risk of bleeding, whereas chronic high-dose aspirin therapy is a more significant contributor to bleeding risk. Further evaluation is needed to confirm these findings.

 The results of our systematic review also suggest that there is no significant difference in the study groups for mortality, MACCE, and thrombosis-related events among the included studies.^10,12^

 Findings from Ndrepepa et al study on aspirin loading doses have raised concerns regarding an increased risk of CNR among patients with acute MI undergoing PCI, while the study’s retrospective observational design and limited sample size may impact the strength of the findings, that warrant further investigation to provide more robust evidence in this area.^13^

###  The Impact of Risk of Bias on Study Outcomes

 The risk of bias was variable among the included studies, with Basili et al^10^ indicating a low risk of bias in all evaluated domains, whereas the observational studies^11,12,13^ had moderate risk across different domains, mainly because of confounding and section bias. However, this moderate risk of bias in the observational studies underscores the importance ofcaution when interpreting findings, particularly those that raise possible safety concerns (i.e. CNR). “Because observational designsremain susceptible to residual confounding, the results of the study may have been impacted by differences in thrombotic risk at baseline, procedure-related factors, and individual differences in aspirin metabolism.

 The present study, like other studies, has some limitations that warrant consideration. One of the main limitations is the limited number of studies evaluating the effects of aspirin reloading, which highlights the need for larger RCTs to better address the current lack of evidence. Additionally, the outcome measures in the included studies were not uniform, making it impossible to conduct a meta-analysis of all outcomes. Finally, due to a limited number of studies we decided to meta-analysis one RCT and one observational study together; however, the I^2^ was 0% indicating the lowest level of heterogeneity among these studies.

## Conclusion

 Based on the available evidence, aspirin loading in patients with CAD who are already on maintenance aspirin therapy appears to be safe and has potential cardiac benefits specifically inhibiting thromboxane B_2_. However, the supporting data are still limited and well-designed RCTs are highly recommended to address this lack of evidence.

## Competing Interests

 The authors declare that there are no conflicts of interest

## Ethical Approval

 Not applicable.

## Supplementary Files

Supplementary file 1 contains Table S1.
